# Morphological Analyses and QTL Mapping of Mottled Leaf in Zucchini (*Cucurbita pepo* L.)

**DOI:** 10.3390/ijms25052491

**Published:** 2024-02-20

**Authors:** Kexin Wang, Xinyu Wang, Lijing Zhang, Yichen Chi, Yusong Luo, Wenlong Xu, Yunli Wang, Shuping Qu

**Affiliations:** 1 Key Laboratory of Biology and Genetic Improvement of Horticultural Crops (Northeast Region), Ministry of Agriculture and Rural Affairs, Northeast Agricultural University, Harbin 150030, China; wkx15104594791@126.com (K.W.); 15765788116@163.com (X.W.); 18804658660@163.com (L.Z.); 18845100319@163.com (Y.C.); yelingyuese@126.com (Y.L.); xwl@neau.edu.cn (W.X.); 2 College of Horticulture and Landscape Architecture, Northeast Agricultural University, Harbin 150030, China

**Keywords:** *Cucurbita pepo* L., QTL analysis, mottled leaf

## Abstract

The mottled leaf is one of the agronomic traits of zucchini and can be applied as a marker trait in aggregation breeding. However, the genetic mechanism responsible for mottled leaf has yet to be elucidated. In the present study, we used two inbred lines (line ‘19’: silver mottled leaf; line ‘113’: normal leaf) as parents for the physiological and genetic analysis of mottled leaf. The synthesis and net photosynthetic rate of chlorophyll were not significantly affected in the mottled areas of leaves. However, we detected a large space between the palisade parenchyma in the leaf mottle area of line ‘19’, which may have caused the mottled leaf phenotype. Light also plays an important role in the formation of mottled leaf, and receiving light during the early stages of leaf development is a necessary factor. Genetic analysis has previously demonstrated that mottled leaf is a quantitative trait that is controlled by multiple genes. Based on the strategy of quantitative trait locus sequencing (QTL-seq), two QTLs were identified on chromosomes 1 and 17, named *CpML1.1* and *CpML17.1*, respectively. Two major loci were identified using R/qtl software version 1.66 under greenhouse conditions in April 2019 (2019A) and April 2020 (2020A) and under open cultivation conditions in May 2020 (2020M). The major QTL, *CpML1.1*, was located in a 925.2-kb interval on chromosome 1 and explained 10.51%-24.15% of the phenotypic variation. The *CpML17.1* was located in a 719.7-kb interval on chromosome 17 and explained 16.25%-38.68% of the phenotypic variation. Based on gene annotation, gene sequence alignment, and qRT–PCR analysis, the *Cp4.1LG01g23790* at the *CpML1.1* locus encoding a protein of the TPX2 family (target protein of Xklp2) may be a candidate gene for mottled leaf in zucchini. Our findings may provide a theoretical basis for the formation of mottled leaf and provide a foundation for the fine mapping of genes associated with mottled leaf. Molecular markers closely linked to mottled leaf can be used in molecular-assisted selection for the zucchini mottled leaf breeding.

## 1. Introduction

Leaf color arises from the dynamic balance of various photosynthetic pigments, including chlorophyll and carotenoids, of which chlorophyll accounts for the largest proportion. Therefore, the color of the leaves is mostly green. When the proportion of photosynthetic pigments in plants changes, healthy leaves will appear in different colors. According to the leaf color phenotype, the color of leaves can be divided into pale green, stripe, spotted leaf, yellow-green, white-green, and other types [1]. According to their anatomical structure, they can be divided into chlorophyll type, air space type, epidermis type, pigment type, and appendage type [2]. Overall, the diversity of leaf colors is beneficial for enhancing the ornamental value of plants. Therefore, some plants intentionally cultivate different leaf color varieties, but usually, only a portion of seeds can transmit traits.

At present, many leaf color mutants have been reported in *Arabidopsis*. The *im* mutant exhibits white-green variation and possesses a small amount of carotenoids, abnormal plastids, and palisade parenchyma in the white area. The *IM* gene encodes a plastid terminal oxidase (PTOX), which participates in the synthesis of carotenoids. The formation of white areas may be due to insufficient carotenoid synthesis in *im* mutants; this can lead to chlorophyll photooxidation and influence the expression of chloroplast genes, ultimately resulting in the formation of leaf variation [3,4,5]. Similar to the *im* mutant, the *var3*, *msl-1*, and *msl-2* mutants also exhibit abnormal palisade tissue [6,7]. Both *var1* and *var2* exhibit a yellow-green-white mottled phenotype. *VAR1* and *VAR2* encode a similar chloroplast FtSH protease, which plays an important role in the degradation of photodamage subunits in photosystem II. *VAR1* and *VAR2* are known to exert synergistic effects. Furthermore, the absence of *VAR1* and *VAR2* may damage the photoprotection mechanism and the development of thylakoids, resulting in variegated leaves [8,9,10,11]. White-green leaf mutants have also been reported in rice. Both *zl16* and *zebra524* exhibited a white-green leaf phenotype, encoding β-hydroxyl ACP dehydratase (HAD) and lycopene β-cyclizing enzymes, respectively, which play an important role in the synthesis of photosynthetic pigments [12,13]. The plastid ribosomal proteins (PRPs) and phosphate ribosamine glycine ligase (PurD) are involved in regulating chloroplast development and chlorophyll metabolism during leaf development to regulate rice leaf color [14,15]. RLK proteins also participate in the whitening of rice leaves through other pathways [16]. Similar phenotypes have also been reported in maize [17].

Among Cucurbitaceae crops, there are the most reports on the leaf color of cucumbers and pumpkins. Yellow-green leaf mutants are commonly found in cucumbers. Both mutants *C777* and *Ygl1* exhibited a yellow-green leaf phenotype. Their candidate genes are involved in chloroplast development and chlorophyll metabolism pathways to regulate cucumber leaf color [18,19]. *Cucurbita pepo* L. is an important commercial crop with good nutritional and medicinal value. *C. pepo* is rich in resources; most germplasm resources possess green leaves, although some plants have yellowing, albinism, or mottled leaves. There are two types of silver mottle on leaves in zucchini; one is a hereditary form of leaf mottle, and the other is caused by whitefly. Hereditary leaf mottle is regulated by a dominant gene which shows an irregular shape and grows along the direction of the leaf vein [20,21,22]. The lower epidermis of the mottled leaves remains green. The expression of the leaf mottle is regulated by the *M* gene, the expression of which can be affected in many ways. Firstly, the *M* gene is regulated by the modified gene, which can affect the expression of *M* in terms of both time and degree. In addition, environmental factors and expression positions play an important role in the expression of the *M* gene. The time at which *M* is first expressed during plant development can also impact the expression levels of the *M* gene [23,24,25]. Furthermore, the *M* gene can cause air space between the palisade parenchyma and the upper epidermal cells, which causes changes in light refraction, making the leaves appear silver. Light reflection from the leaf mottle area has been shown to range from 400 to 700 nm, which is higher than that of the green area of leaves [26]. In addition to hereditary leaf mottle, leaf mottle can also be caused by whitefly, which can be affected by light, temperature, humidity, and insect density. Chloroplasts in palisade cells and the plasma membrane around some vascular cells exhibited slight ultrastructural damage, thereby affecting photosynthesis and the agronomic traits of plants [27,28]. The structures of hereditary leaf mottle and the leaf mottle caused by whitefly are similar, with spaces between the palisade cells and the upper epidermis. However, few studies have investigated the location of mottled leaf in *C. pepo*. A genetic linkage map of *C. pepo* was constructed using BC_1_ and showed that the dominant gene *M* was located in the linkage group 6 [29]. Through a high-density genetic map of *C. pepo* using an RIL population, a major QTL related to leaf mottle was located in a 0.29 Mb interval on chromosome 17 that explained 23.3% of the phenotypic variation. Two minor QTLs were also found to be located on chromosome 1 (with a 0.18 Mb interval) and chromosome 13 (with a 1.67 Mb interval); these explained 3.83% and 8.01% of the phenotypic variation, respectively [30].

In this study, we used lines ‘19’ and ‘113’ as materials to observe mottled leaf phenotype, constructed F_2_ populations for QTL analysis and stability analysis, and analyzed the interaction of mottled leaf QTLs. Our findings indicate that molecular markers closely linked to mottled leaf can be used for molecular marker-assisted selection breeding in zucchini and provide a foundation for the fine mapping of genes and the molecular mechanistic research of mottled leaf.

## 2. Results

### 2.1. Observation of the Phenotypes Associated with the Mottled Leaf Trait

**Figure 1:**
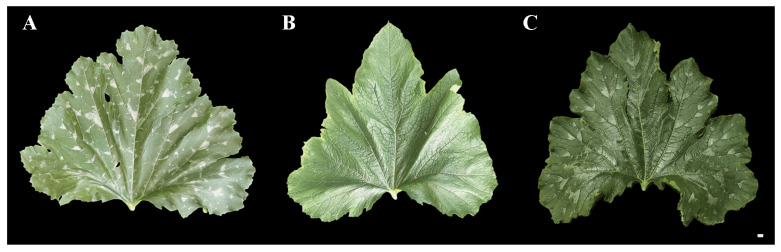
The phenotypes of ‘19’, ‘113’ and F_1_. (**A**) 19. (**B**) 113. (**C**) F_1_. Scale = 1 cm.

We observed the mottled leaf traits of lines ‘19’ and ‘113’ during growth and observed that leaf mottle appeared on the leaves of line ‘19’ during the 8-leaf stage. There was no significant leaf mottle at this stage, although chlorosis began on the first and second leaves under the growing tip. Subsequently, the area of chlorosis gradually turned silver, and leaf mottle was formed until the fifth leaf stage under the growing tip. As the plant grew, the leaf mottle became more prominent. Subsequently, all leaves were covered with leaf mottle. No chlorosis and leaf mottle appeared in line ‘113’ during the growth period. The appearance period of leaf mottle in the F_1_ generation was similar to that of line ‘19’, although the grade of leaf mottle was lower compared to line ‘19’ (Figure 1). The mottled leaf phenotype was most significant at the 20-leaf stage. Lines ‘19’ and ‘113’, and the F_1_ generation, were classified as grade 3, grade 0, and grade 2, respectively.

### 2.2. Determination of the Content of Photosynthetic Pigment in Leaves

In order to investigate the changes in pigment content, we selected the mottled area and green area of parent ‘19’ and the corresponding position of ‘113’ at the 20-leaf-stage to determine pigment content. 

**Figure 2:**
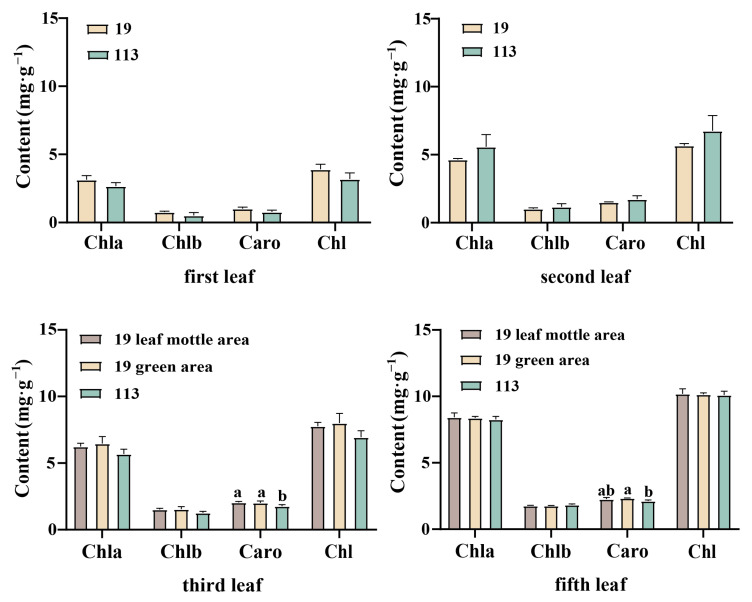
Photosynthetic pigment content of parent in different stages. Chl a: chlorophyll a, Chl b: chlorophyll b, Caro: carotenoid, Chl: chlorophyll. Different letters indicate significant differences at 0.05 level. Columns are the mean values ± SD. Each column has been tested in three replicates.

The first, second, third, and fifth leaves under the growing tip at the 20-leaf-stage were selected for the determination of pigment content. The color of the first and second leaves of parent ‘19’ was an inconspicuous chlorotic mottle. The pigment content of all the leaves was measured collectively. Leaf mottle was evident on the third and fifth leaves, and the pigment content was measured separately for the green and mottle areas. The content of chlorophyll a in the two parents was always higher than that of chlorophyll b, with a ratio between 4.1 and 5.7. The ratio of carotenoid to total chlorophyll was stable between 0.22 and 0.27. Due to the higher chlorophyll content of the two parents, the leaves were predominantly green. Except for the significantly higher carotenoid content in the green area of line ‘19’ of the third and fifth leaves when compared to the corresponding area of line ‘113’, there was no significant difference in chlorophyll content and carotenoid content between the two lines of plants (Figure 2). There was no significant difference in the levels of chlorophyll a, chlorophyll b, carotenoid, and total chlorophyll in the first, second, third, and fifth leaves of the mottled area in line ‘19’ and the green area in line ‘19’. Moreover, there was no significant difference in the chlorophyll content when comparing the mottle area and the green area in line ‘19’, thereby indicating that the formation of leaf mottle did not affect the synthesis of carotenoid and chlorophyll.

### 2.3. Analysis of Photosynthetic Parameters in Leaves

**Figure 3:**
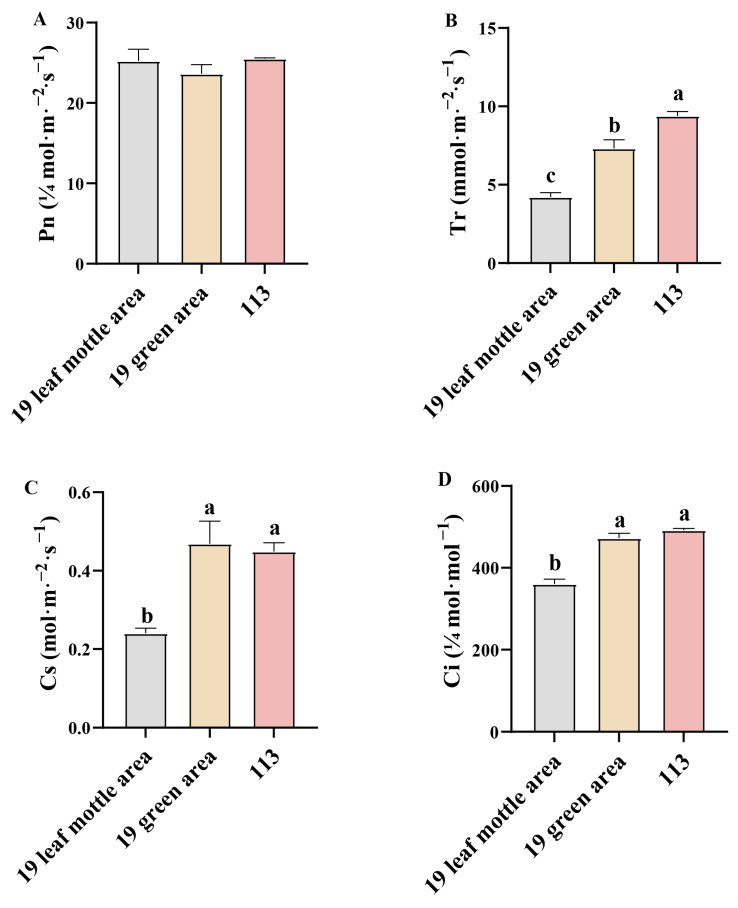
Photosynthetic parameters in different leaf areas. (**A**) net photosynthetic rate of parents. (**B**) Transpiration rate of parents. (**C**) Stomatal conductance rate of parents. (**D**) Intercellular CO_2_ concentration rate of parents. Different letters indicate significant differences at 0.05 level. Columns are the mean values ± SD. Each column has been tested in three replicates.

Next, we determined the net photosynthetic rate (Pn), transpiration rate (Ti), stomatal conductance (Cs), and intercellular CO_2_ concentration (Ci) of lines ‘19’ and ‘113’ at the 20-leaf-stage. Analysis showed that there was no significant difference in net photosynthetic rate between the two parents (Figure 3A). Compared with the green area of lines ‘19’ and ‘113’, the Ti, Cs, and Ci of the mottle area in line ‘19’ were all significantly reduced (Figure 3B–D). The formation of leaf mottle, therefore, affects Ti, Cs, and Ci but has no significant impact on Pn.

### 2.4. Shading Analysis of Leaf

**Figure 4:**
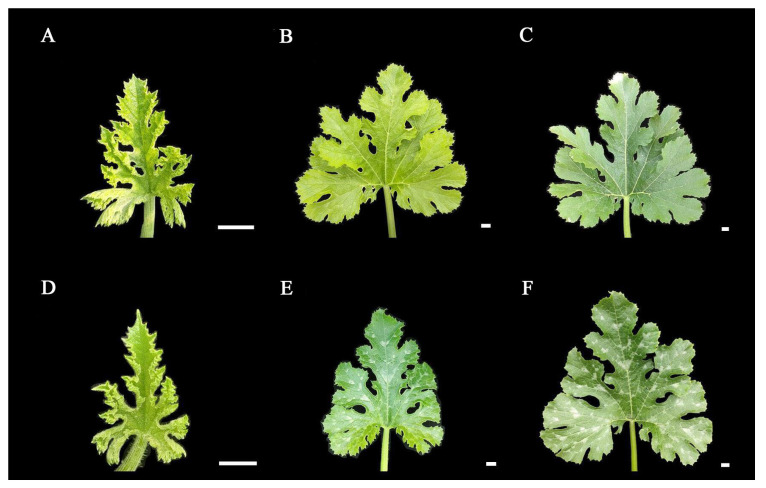
Results of leaves under shading treatments. (**A**) The leaf without shading treatment. (**B**) The leaf under 5-days of shading. (**C**) The leaf under 5-days of shading, then 10-days of light restoration. (**D**–**F**) Leaves growing under light in the same periods as (**A**–**C**). Scale = 1 cm.

In order to investigate whether light exerts an impact on the formation of leaf mottle, we applied shading treatment to the leaves of line ‘19’. We selected plants with the same extent of growth, covered the first true leaf under the growing tip, and plants with all leaves exposed to natural light acted as controls. Analysis showed that no leaf mottle appeared on the leaves under the 5-days shading treatment. Subsequently, the cover was removed, and all leaves were exposed to natural light. After 10-days, no mottle had appeared on the leaves (Figure 4A–C). The control group maintains a mottled leaf phenotype during these stages (Figure 4D–F). Our findings indicate that light is involved in the formation of leaf mottle, and that receiving light during the early stages of leaf development is a necessary factor for the formation of leaf mottle.

### 2.5. Analysis of Differences in the Microstructure of Leaves 

**Figure 5:**
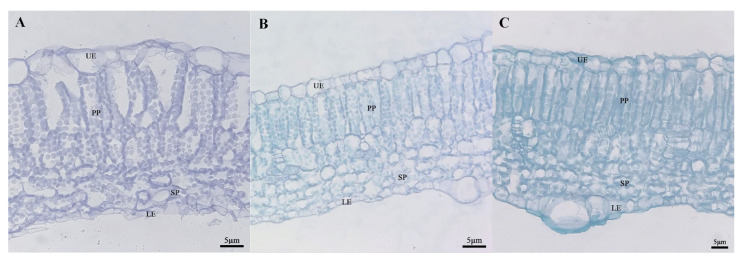
The cross-sectional structure of parental. (**A**) Silver area of ‘19’ (40×). (**B**) Green area of ‘19’ (40×). (**C**) ‘113’ (40×). UE: upper epidermis; PP: palisade parenchyma; SP: sponge parenchyma; LE: Lower epidermis. Scale = 5 μm.

In order to determine whether there was a structural difference between the mottled area and the green area of leaves, we selected the fifth leaf (a mature and functional leaf) under the growing tip at the 20-leaf-stage for analysis. At this time, leaf mottle had developed obviously on the leaves. The green area and mottle area of line ‘19’ and the corresponding area of line ‘113’ were selected to prepare paraffin-embedded sections. The zucchini leaves were composed of an upper epidermal layer, a palisade parenchyma, a sponge parenchyma, and a lower epidermal layer (Figure 5). In the green area of line ‘19’ and line ‘113’, the palisade parenchyma was closely arranged (Figure 5B,C), while the palisade parenchyma in the mottled area of leaves from line ‘19’ was loosely arranged and contained large air space between cells. The sponge parenchyma in the green area of the leaves from line ‘19’ and line ‘113’ was tighter than in the mottled area on leaves from line ‘19’ (Figure 5). Next, we measured the leaf thickness and the cell thickness of each layer of the two parents. Analysis showed that the thickness of cells in the upper epidermis cells and the sponge parenchyma in line ‘19’ exhibited significant differences when compared between the mottle area and green area (Table 1).

### 2.6. Genetic Analysis of Mottled Leaf Trait

**Figure 6:**
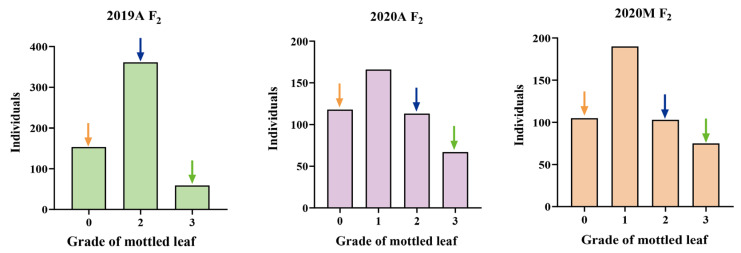
Frequency histogram of leaf mottle grades in different F_2_ populations. The orange arrows indicate mottled leaf phenotype in ‘19’, the green arrows indicate mottled leaf phenotype in ‘113’, and the blue arrows indicate mottled leaf phenotype in F_1_.

F_1_ populations derived by crossing lines ‘19’ and ‘113’ showed significant leaf mottle on their leaves (grade 2) (Figure 1). An F_2_ generation was constructed by F_1_ self-crossing. The leaf mottle grades of the F_2_ population planted in different environments were divided into three grades (grade 0, grade 2, and grade 3) for 2019A and four grades (grade 0, grade 1, grade 2, and grade 3) for 2020A and 2020M. It was found that the absolute values of skewness and kurtosis of mottled leaf traits in the F_2_ population under different environments were <1 (Table S1), and the population phenotypic distribution conformed to a typical normal distribution (Figure 6). Therefore, the mottled leaf trait was a quantitative trait.

### 2.7. QTL Analysis of the Mottled Leaf Trait

**Figure 7:**
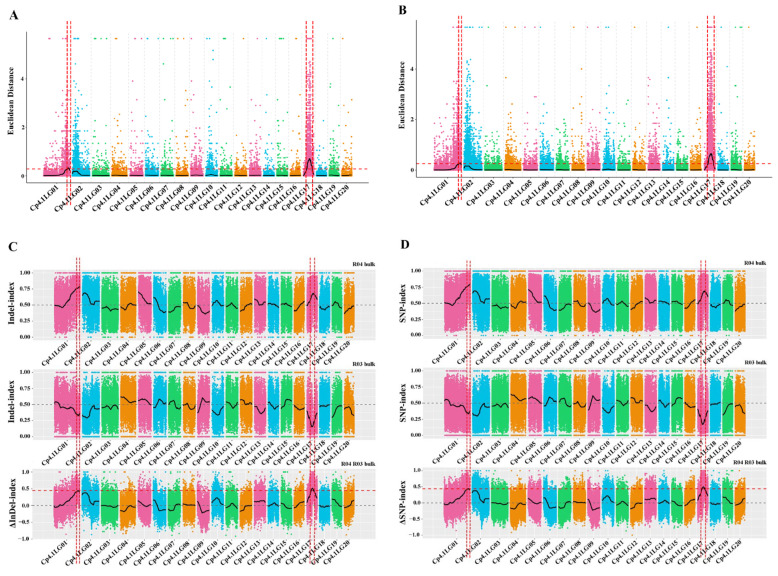
The results of QTL-seq. (**A**) Distribution of the ED-based linkage value with InDels on chromosomes. (**B**) Distribution of the ED-based linkage value with SNPs on chromosomes. (**C**) Distribution of InDel-index correlation values on chromosomes. (**D**) Distribution of SNP-index correlation values on chromosomes. In (**A**,**B**), the medians +3 SD of the fitted value of all sites were defined as the linkage threshold and shown by red lines. In (**C**,**D**), the top panel is a distribution map of the SNP-index (InDel-index) values of the M-pool, the middle panel is a distribution map of the SNP-index (InDel-index) values of the N-pool, and the bottom panel is a distribution map of the Δ(SNP-index)/Δ(InDel-index) values. The red line represents the confidence threshold line for the 99 percentile.

In total, 49,104,069 clean reads and 49,745,164 clean reads were obtained from the M-pool (28× read depth with 97.21%) and the N-pool (28× read depth with 97.22%) using QTL-seq. Q30 values reached 88.18% and 87.99%, respectively. These results indicated that the sequencing results were reliable for gene mapping. A total of 979,650 SNPs and 324,752 InDels were identified on all 20 chromosomes. SNPs/InDels with multiple genotypes, a read depth <4 in both pools, or consistent genotypes in both pools were removed, thus leaving 617,314 high-quality SNPs, and then 191,073 high-quality InDels were obtained. Next, the ED algorithm was employed to identify significantly different InDels between the M-pool and N-pool based on sequencing data to predict candidate regions of the mottled leaf. A 1.26-Mb region and a 4.30-Mb region were identified on chromosomes 1 and 17, respectively (Figure 7A). The ED algorithm was also employed to identify significantly differential SNPs between the M-pool and N-pool based on sequencing data to predict candidate regions of the mottled leaf. A 1.35-Mb region and a 4.35-Mb region were identified on chromosomes 1 and 17, respectively (Figure 7B). The Δ(InDel-index) and Δ(SNP-index) were calculated and plotted by comparing the results of the InDel-index and SNP-index of the M-pool and N-pool in the genomic positions. The threshold of the Δ(InDel-index) value was 0.45; only one region was found on chromosome 17 (total length 2.10 Mb) (Figure 7C). The threshold of the Δ(SNP-index) value was 0.44, and two regions were found on chromosome 1 (total length 0.06 Mb) and chromosome 17 (total length: 2.03 Mb), respectively (Figure 7D). Combining the results of these analyses, the candidate regions associated with the mottled leaf trait were positioned within the 19.71–20.97 Mb intervals on chromosome 1 and between the 3.83–6.14 Mb intervals on chromosome 17. Therefore, we named these loci as *CpML1.1* and *CpML17.1*, respectively.

**Figure 8:**
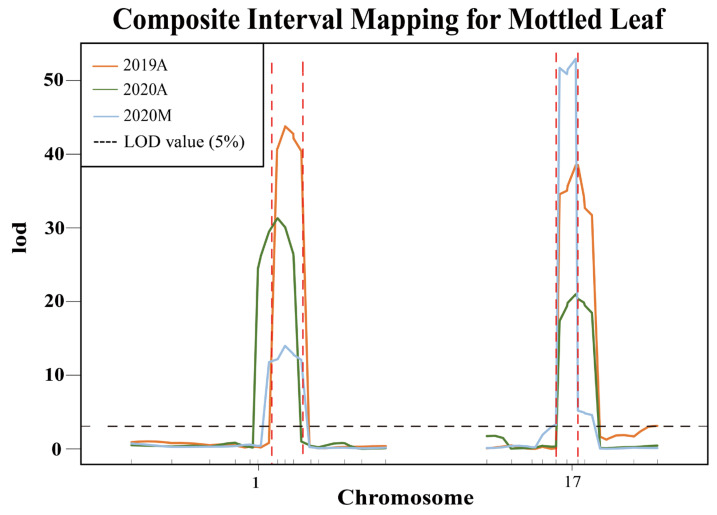
Verification of QTL stability. The area between the red dashed lines represents the QTL location.

Based on the InDels in the candidate region between parental genomes, 25 polymorphic markers were developed on chromosomes 1 and 17 (Table S2). To test the stability of the QTLs *CpML1.1* and *CpML17.1*, we analyzed 580 F_2_ plants in 2019A, 580 F_2_ plants in 2020A, and 480 F_2_ plants in 2020M. R/qtl analysis showed that a total of two QTLs associated with the mottled leaf trait were detected on chromosomes 1 and 17 (Figure 8). In 2019A, 2020A, and 2020M, *CpML1.1* was located between markers Chr01_18713051 and Chr01_19638223, Chr01_18590758 and Chr01_19419173, Chr01_18713051 and Chr01_19638223, respectively, with LOD scores of 43.79, 31.18, and 14.02. These scores explained 24.15%, 23.82%, and 10.51% of the phenotypic variation. With regards to the intersection of associated regions from QTL mapping, we used the CIM method in the three environments and QTL-seq analysis and determined that *CpML1.1* was located in a 925.2-kb region between markers Chr01_18713051 and Chr01_19638223. *CpML17.1* was located between markers Chr17_5225091 and Chr17_5944838, Chr17_5225091 and Chr17_5944838, Chr17_5225091 and Chr17_5944838, respectively, with LOD scores of 38.70, 20.65 and 52.99, thereby explaining 20.80%, 16.25%, and 38.68% of phenotypic variation. Combining the mapping results of different populations, *CpML17.1* was located in the 719.7-kb region between markers Chr17_5225091 and Chr17_5944838 (Table 2).

### 2.8. QTL Interaction Analysis of Mottled Leaf Trait

**Figure 9:**
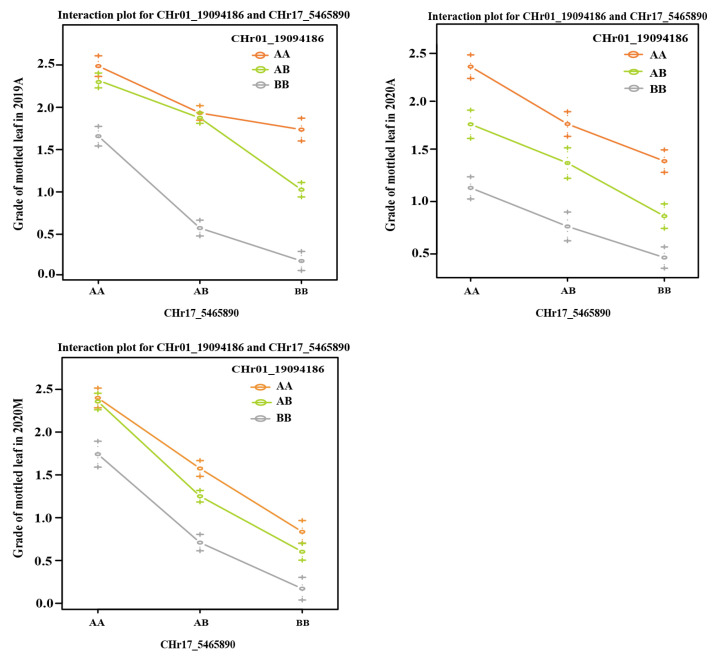
Interaction plots of the QTL pairs detected in the F_2_ and RIL populations for mottled leaf.

Both *CpML1.1* and *CpML17.1* exhibited a positive additive effect on mottled leaf traits. In order to study the interaction effect of *CpML17.1* and *CpML1.1*, we analyzed the genotype and phenotype of the two loci (Figure 9). When both loci carry homozygous ‘A’ (from mottled leaf material from line ‘19’), the grade of mottled leaf was the highest, and when both loci carried the homozygous ‘B’ (from no-mottled-leaf material from line ‘113’), the grade of mottled leaf was the lowest. Under different environmental conditions, in *CpML1.1 BB* plants (the *CpML1.1* locus genotype was *BB*), the mottled leaf grade was *CpML1.1 AA* > *CpML1.1 AB* > *CpML1.1 BB*. In *CpML17.1 BB* plants (the *CpML17.1* locus genotype was *BB*), the mottled leaf grade was *CpML17.1 AA* > *CpML17.1 AB* > *CpML17.1 BB*. These results showed that *CpML1.1* and *CpML17.1* exerted cumulative effects on mottled leaf traits.

### 2.9. Candidate Gene Analysis of Major QTLs

According to the Cucurbit Genomics Database and SoftBerry, a total of 105 putative genes were identified in the *CpML1.1* region (925.2 kb) and 41 putative genes in the *CpML17.1* region (719.7 kb). Of these, 12 genes in the *CpML1.1* region and 15 genes in the *CpML17.1* region had non-synonymous changes in the CDS region and InDels/SNPs in the promoter element when comparing lines ‘19’ and ‘113’ (Tables S3 and S4). In addition, we found that changes in the arrangement of palisade cells were the main reason underlying the mottled leaves. Therefore, candidate genes may be involved in the development of palisade cells. In addition, previous research reported that E3 ubiquitin ligase also participates in the formation of air-space type leaf color mutants [31]. Therefore, we first selected genes related to cell development and encoding E3 ubiquitin ligase for further validation. *Cp4.1LG17g08140* encodes an E3 ubiquitin ligase, while *Cp4.1LG17g08260* encodes a TBL protein that has been reported to participate in the growth and development of cell walls. *Cp4.1LG17g08300* encodes a beta-glucosidase, which represents one of the important components of cellulase that is mainly involved in cell wall degradation. *Cp4.1LG01g23790* encodes a TPX2 family protein (target protein of Xklp2), which is a microtubule-associated protein. The functions of these three genes are all related to cellular development processes. We compared the protein sequence differences of these four genes in the parents, and all four genes have non-synonymous mutations (Figures S1–S4).

**Figure 10:**
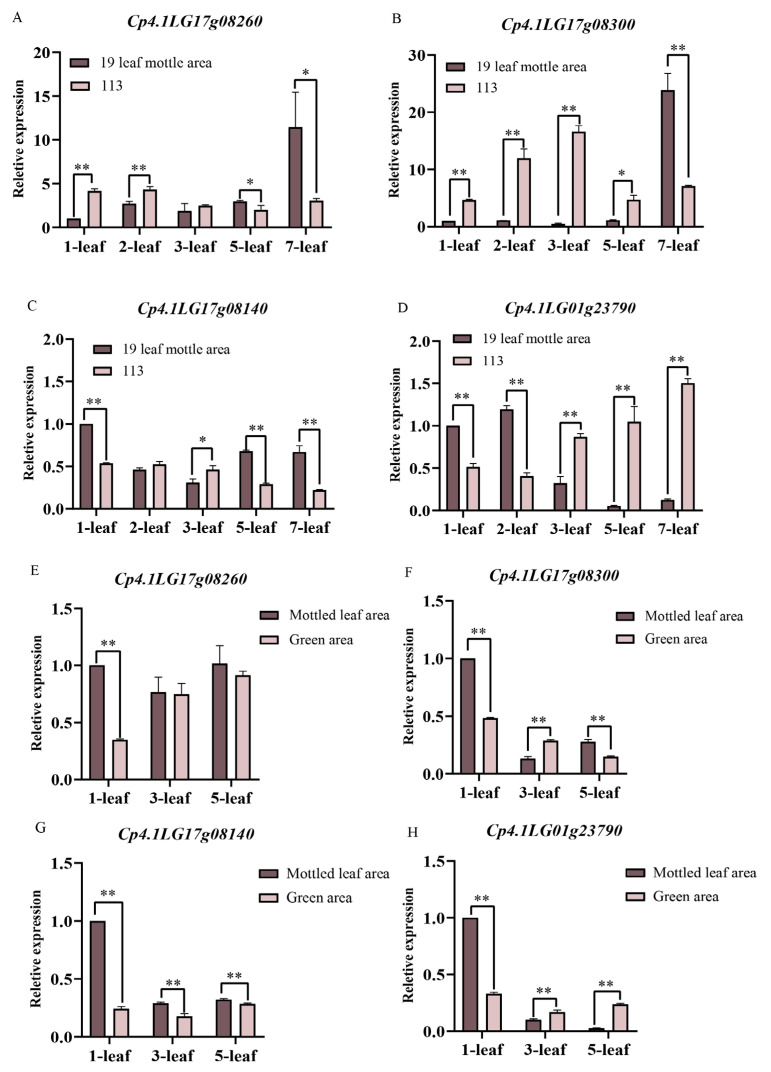
Relative expression levels of four candidate genes in the ‘19’ and ‘113’ and F_2_ populations. (**A**) Relative expression levels of *Cp4.1LG17g08260*. (**B**) Relative expression levels of *Cp4.1LG17g08300*. (**C**) Relative expression levels of *Cp4.1LG17g08140*. (**D**) Relative expression levels of *Cp4.1LG01g23790*. (**A**–**D**) Relative expression levels of four candidate genes in ‘19’ and ‘113’. (**E**) Relative expression levels of *Cp4.1LG17g08260*. (**F**) Relative expression levels of *Cp4.1LG17g08300*. (**G**) Relative expression levels of *Cp4.1LG17g08140*. (**H**) Relative expression levels of *Cp4.1LG01g23790*. (**E**–**H**) Relative expression levels of four candidate genes in the F_2_ populations. The relative expression levels of four genes were quantified using the 2^−ΔΔCT^ method. For the two parents, the relative expression level at the 1-leaf-stage in ‘19’ was set to a value of 1 and used as a reference, respectively. For the F_2_, the relative expression level at the 1-leaf-stage in mottled leaf area was set to a value of 1 and used as a reference, respectively. ** indicates an extremely significant difference, *p* < 0.01; * indicates a significant difference, *p* < 0.05.

We selected parental and F_2_ leaves for qRT-PCR. The expression levels of *Cp4.1LG17g08260* in the first and second leaves of line ‘19’ were significantly lower than those of line ‘113’. An opposite trend of expression was observed for *Cp4.1LG17g08260* in the fifth and seventh leaves. In the F_2_ population, the expression levels of the *Cp4.1LG17g08260* only showed a significant difference in the leaf mottle area and green area during the first leaf period (Figure 10A,E). During the first to fifth leaf period, *Cp4.1LG17g08300* was only expressed at a low expression level in the leaf mottle area of line ‘19’ and was significantly lower than in line ‘113’. However, during the seventh leaf period, the expression level of *Cp4.1LG17g08300* suddenly increased in the leaf mottle area of line ‘19’, significantly higher than the level detected in line ‘113’. In the F_2_ population, the expression level of *Cp4.1LG17g08300* in the leaf mottle area showed a gradually decreasing trend, and the expression level in the leaf mottle area was higher than that in the green area during the first and fifth leaf periods (Figure 10B,F). The expression level of *Cp4.1LG17g08140* was lower than line ‘113’ in the second and third leaves of line ‘19’ and significantly higher than line ‘113’ in the other periods. The trend in expression levels of *Cp4.1LG17g08140* in the F_2_ population was opposite to the parents in the third leaves (Figure 10C,G). The expression level of *Cp4.1LG01g23790* was significantly higher than line ‘113’ in the first and second leaf periods of line ‘19’ but significantly lower than line ‘113’ in the third, fifth, and seventh leaf periods. In the F_2_ population, *Cp4.1LG01g23790* was expressed at higher levels in the leaf mottle area during the first leaf period and at lower levels during the third and fifth leaf periods (Figure 10D,H).

Thus, our analysis revealed that the expression trends for *Cp4.1LG17g08260*, *Cp4.1LG17g08300*, and *Cp4.1LG17g08140* between the leaf mottle area and green area in the F_2_ population were opposite to those of their parents. The expression trend for *Cp4.1LG01g23790* followed the same trend as their parents. Therefore, we speculated that *Cp4.1LG01g23790* is a possible candidate gene that plays a key role in the formation of mottled leaves.

## 3. Discussion

Various factors are responsible for leaf color variation and can be divided into the pigment type and non-pigmented type. Most leaf color mutations are mainly caused by changes in chlorophyll, carotenoid, and anthocyanin content. In the present study, we found that silver mottle appeared on zucchini leaves. To determine whether the appearance of the mottled leaf is related to chlorophyll and carotenoids, we measured the content of the photosynthetic pigment in the leaves of zucchini. The chlorophyll contents of the lines ‘19’ and ‘113’ were not significantly different. The differences in photosynthetic rate between these three areas were not significant. Therefore, the formation of leaf mottle in ‘19’ does not affect chlorophyll content or chloroplast development. This indicates that mottled leaf may not affect yield. In addition, we constructed nearly isogenic lines (NILs) for the two QTL loci *CpML1.1* and *CpML17.1* identified in the article, using line ‘113’ as the recurrent parent—both NIL-ML1.1 and NIL-ML17.1 exhibit mottled leaf phenotype. We simultaneously planted NIL-ML1.1, NIL-ML17.1, and recurrent parent ‘113’ for unified field management. Compared with ‘113’, the number of fruits and single fruit weight of NIL-ML1.1 and NIL-ML17.1 did not significantly decrease. This result further confirms this speculation.

The morphology analysis of mottled leaves and green leaves showed that the palisade tissue was closely arranged in the green area of line ‘19’ and ‘113’, but was loosely arranged and featured air space between cells in the mottled area of leaves from line ‘19’. Therefore, the air space between the palisade tissue in the leaf mottle area of line ‘19’ may be the main reason for the mottled leaf phenotype. Our findings are similar to those of Scarchuk, who previously reported the presence of air space between the palisade cells from the leaf mottle area and that these cells were not tightly associated with epidermal cells. These air spaces were found to be responsible for the silver leaf mottle phenotype [32]. A similar phenomenon has also been reported in ornamental plants. Air spaces between the cells were reported to cause leaf variegation, confirmed in *Erythronium dens-canis* L. and *Egonia rexPutz* [33,34]. In the present study, we found that the formation of mottled leaf requires the participation of light. However, the growth of leaf mottle varied between parental materials grown in different environments. Therefore, the formation of mottled leaf may be related to factors such as light quality, light intensity, and light duration. In Cucurbitaceae crops, the formation of mottled leaves in cucumber is also light-dependent and is most sensitive to ultraviolet light in sunlight [35]. In the future, we plan to investigate the effects of different light qualities on the formation of mottled leaf in zucchini to explore the most suitable light environment for the growth of mottled leaf in zucchini.

QTL-seq and the detection of molecular markers reveal that the silver leaf gene was located at an interval of 287.15 kb on chromosome 4 in *Cucurbita moschata*. The segregation ratio of green leaf to silver leaf was 3:1, showing that the silver leaf trait was inherited in a completely recessive manner. The gene *CmoCh04G023390* gene was located in the candidate interval of chromosome 4 in *C. moschata* and can be mapped to chromosome 1 in zucchini at the physical position of 16561706 bp to 16565740 bp. A major QTL for *Cucurbita pepo* L. silver leaf *Sl12_1* was detected on chromosome 17 at a physical position of 1.72 Mb-2.01 Mb. Two minor QTLs for silver leaf *Sl1_1* and *Sl16_1* were detected on chromosome 1 and chromosome 13 at the physical locations of 0.54 Mb-0.72 Mb and 0.1 Mb-1.77 Mb, respectively [30]. In our present research, we found that the mottled leaf trait was a quantitative trait. Two major QTLs were detected using composite interval mapping. *CpML1.1* was located on chromosome 1 at a physical position of 18.7 Mb-19.6 Mb. *CpML17.1* was located on chromosome 17 at a physical position of 5.22 Mb-5.94 Mb. Compared with previous results, *Sl1_1* and *CpML1.1* were both located on chromosome 1, while *Sl12_1* and *CpML17.1* were both located on chromosome 17. However, the physical positions of the two QTLs identified in this study were located far from *Sl1_1* and *Sl12_1*. Therefore, we hypothesize that *CpML1.1* and *CpML17.1* may represent two new QTLs that control mottled leaf.

**Figure 11:**
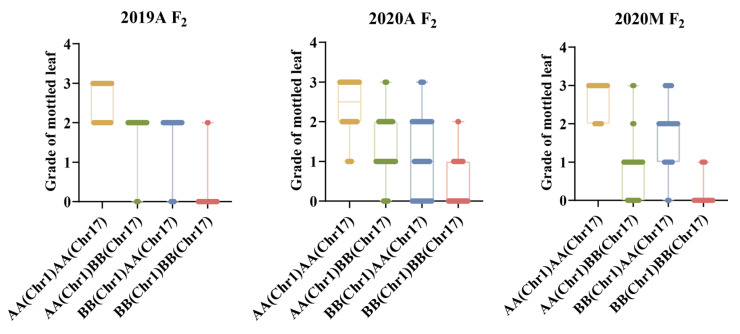
Results of the mottled leaf grade when *CpML1.1* and *CpML17.1* carry different homozygotes.

To verify the accuracy of the QTL-seq results, we developed InDel molecular markers in the candidate regions to test the stability of the loci and narrow the location intervals. We found that the inheritance of *CpML1.1* and *CpML17.1* were stable in different environments. In different environments, when both loci carry a homozygous ‘A’, the mottled leaf is classified as grade 2–3. When both loci carry a homozygous ‘B’, the grade of the mottled leaf was 0–1 grade. When one of the two loci was homozygous for ‘A’, the phenotype of the mottled leaf was grade 1–3, which indicates that the two loci have an additive effect on the formation of leaf mottle. The more homozygote ‘A’ carried, the higher the grade of the mottled leaf. In 2019A, the *CpML1.1* and *CpML17.1* explained 24.15% and 20.80% of the phenotypic variation, respectively. When *CpML1.1* and *CpML17.1* carry different homozygosity, the mottled leaf grade was concentrated in grade 2, and the influence between the two loci is not significant. In 2020A, when *CpML1.1* and *CpML17.1* exhibited different homozygosity, the highest grade of mottled leaf was grade 2. However, when *CpML1.1* was homozygous for ‘B’, and *CpML17.1* was homozygous for ‘A’, there were more plants that were grade 0. The phenotypic variation of *CpML1.1* was also higher than that of *CpML17.1*. Therefore, the effect of *CpML1.1* was slightly stronger than that of *CpML17.1*. In 2020M, when *CpML1.1* was homozygous for ‘A’ and *CpML17.1* was homozygous for ‘B’, the mottled leaf grade was mainly concentrated in grades 0–1. When *CpML1.1* was homozygous for ‘B’, and *CpML17.1* was homozygous for ‘A’, the mottled leaf grades were mainly concentrated in grades 1–2. In this environment, the LOD score for *CpML17.1* was as high as 52.99, explaining the phenotypic variation of 38.68%. *CpML1.1* explained 10.51% of the phenotypic variation. This phenomenon indicates that *CpML17.1* plays a stronger role than *CpML1.1* (Figure 11). Thus, the strength of *CpML1.1* and *CpML17.1* will be affected in different environments, but both loci will exert their effectiveness. Due to the limitations of F_2_ population mapping, it is impossible to locate a single QTL precisely. Many quantitative traits are currently mapped by applying the NIL method. In order to investigate the relationship between *CpML1.1* and *CpML17.1* further, it was necessary to construct NILs for the two QTLs, respectively. These two NILs can be used for fine mapping and provide a foundation for gene cloning.

Cell division is crucial for plant growth and development, and microtubules are essential for eukaryotic cell division, expansion, and differentiation [36,37]. Plants have unique microtubule arrays that control the direction of cell division and expansion, mainly regulated by microtubule-associated proteins (MAPs). There are various conserved microtubule-related proteins in eukaryotes, such as augmin, TPX2, CLASP and EB1. By applying gene annotation, gene sequence alignment, and qRT-PCR analysis, we identified a *Cp4.1LG01g23790* gene, which encodes a TPX2 family protein (target protein of Xklp2). TPX2 (target protein Xklp2) is an evolutionarily conserved microtubule-associated protein and a key factor for mitotic spindle assembly factor. To date, all plant TPX2-family proteins have been shown to bind to microtubules and function in distinct processes such as cell division and the regulation of hypocotyl cell elongation by hormones and light signals [38,39]. Thus far, several TPX2 family proteins have been reported, including TPX2, WAVE DAMPENED 2 (WVD2), WAVE DAMPENED 2 LIKE (WDL) 1, 2, and 3 and MAP20. In *Arabidopsis*, WAVE-DAMPENED2-LIKE5 (WDL5) is a microtubule-stabilizing protein that plays a positive role in ethylene-regulated hypocotyl cell extension [40]. The overexpression of *EgMAP20* and *EgWDL3L* in *Arabidopsis* leads to changes in cell morphology and results in organ-twisting phenotypes [41]. However, there have been no reports on how TPX2 could influence the arrangement of leaf cells. It has been reported that the microtubule protein CLASP can influence the arrangement of leaf cells. In *Arabidopsis*, the *clasp-1* mutant exhibits defects in cell-directed amplification [42]. The lack of microtubule-related protein CLSAP leads to changes in cell division patterns, resulting in significant distortions in the topological relationships between cells and intercellular spaces and changes in their relative abundance [43]. In the present study, we detected a difference in the expression of TPX2 between the mottled area and green area; this may influence cell division and lead to mottled leaf.

## 4. Materials and Methods

### 4.1. Plant Material

The two *C. pepo* inbred lines ‘19’ and ‘113’ were originally developed by the Laboratory of Pumpkin Molecular Genetic Breeding, Northeast Agricultural University, Harbin, China. Inbred line ‘19’ showed significant silver mottle on leaves; there was no leaf mottle on inbred line ‘113’. The F_1_ and F_2_ populations derived by crossing ‘19’ and ‘113’ were constructed to analyze the inheritance and QTL analysis for mottled leaves. 40 ‘19’, 40 ‘113’, 40 F_1_, and 580 F_2_ were planted in a greenhouse in April 2019 (2019A). 40 ‘19’, 40 ‘113’, 40 F_1_, and 580 F_2_ were planted in a greenhouse in April 2020 (2020A). 40 ‘19’, 40 ‘113’, 40 F_1_, and 480 F_2_ were planted under open cultivation in May 2020 (2020M). All plants were grown at the Xiangyang Base of Northeast Agricultural University, Harbin, China (N45°77′, E126°92′). 

### 4.2. Determination of Photosynthetic Pigments

The first, second, third, and fifth leaves from the growing tip were selected as the material during the 20-leaf-stage. The mottle area and green area of ‘19’ and the green leaves in the same area for ‘113’ were collected. The pigment in the leaves was extracted using 80% acetone. The absorbance of the extract was measured at 663, 645, and 470 nm using a microplate reader. The control was 80% acetone. Three biological repeats were determined for each material, and technical repeats were performed three times in each biological repeat. The formula for calculating pigment content is as follows:

C_chla_ (mg/g) = 12.21 × A663 − 2.81 × A645

C_chlb_ (mg/g) = 20.13 × A645 − 5.03 × A663

C_caro_ (mg/g) = (1000 × A470 − 3.27 × Ca – 104 × Cb)/229

Total Chl = C_chla_ + C_chlb_

### 4.3. Determination of Photosynthetic Parameters

The fifth true leaf from the growing tip of the 20-leaf-stage was selected to measure the photosynthetic parameters. The mottle area and green area of ‘19’ and the green leaves in the same area for ‘113’ were collected. Net photosynthetic rates (Pn; µmol CO_2_ m^−2^s^−1^), stomatal conductance (Gs; mol H_2_O m^−2^s^−1^), intercellular carbon dioxide concentrations (Ci; µmol CO_2_ mol^−1^), and transpiration rates (Ts; mmol H_2_O m^−2^s^−1^) were measured, using CI-340. Three biological repeats were determined for each material, and technical repeats were performed three times in each biological repeat.

### 4.4. Leaf Anatomy Assay

At the stage of twenty leaves, the fifth leaf under the growing tip is used for leaf structure analysis. The mottle area and green area of ‘19’ and the green leaves in the same area for ‘113’ were collected. Prepare FAA and different concentrations of alcohol to fix and dehydrate the leaves and dye them with safranin-fast green. After taking photos, use ImageJ to measure the thickness of the upper epidermis, palisade parenchyma, sponge parenchyma, and lower epidermis of leaves.

CTR = (thickness of palisade parenchyma/leaf thickness) × 100%

SR = (thickness of sponge parenchyma /leaf thickness) × 100%

### 4.5. Inheritance Analysis of the Mottled Leaf Trait

**Figure 12:**
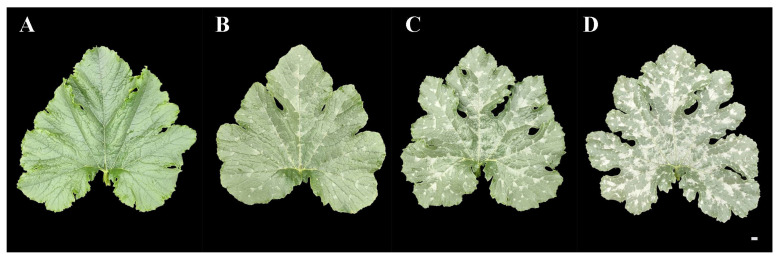
Grading standard of F_2_ mottled leaf. (**A**) 0 grade. (**B**) 1 grade. (**C**) 2 grade. (**D**) 3 grade. Scale = 1 cm.

F_2_ population of ‘19’ and ‘113’ was constructed for inheritance analysis of the mottled leaves. We conducted a survey on mottled leaf grade during the 20-leaf-stage. According to the percentage of mottled area to total leaf area, the grade of mottled leaf was divided into three grades in 2019 and four grades in 2020. The grade 0 plant showed no leaf mottle. The leaf mottle area of grades 1 to 3 accounts for 20%, 50%, and 80% of the total leaf area, respectively (Figure 12).

### 4.6. Pool Construction and QTL-seq Analysis

Genomic DNA was extracted from ‘19’, ‘113’, F_1,_ and F_2_ using the modified cetyltrimethylammonium bromide (CTAB) method. The mottled leaf pool (M-Pool) and no-mottled-leaf pool (N-Pool) were constructed by mixing equal amounts of DNA from 30 extremely mottled leaf plants (grade 3) and 30 extremely without mottled leaf plants (grade 0) from the 2019A F_2_ population. The parental DNA pools were constructed by mixing equal amounts of DNA from the 30 ‘19’ and 30 ‘113’ plants. DNA libraries were sequenced on the Illumina Hiseq 2000 platform in BioMaker (Peking, China). In order to ensure the quality of information analysis, the Raw reads were filtered to obtain clean reads. The clean reads of all samples were compared with the reference genome of zucchini (http://cucurbitgenomics.org/organism/14, accessed on 2 July 2019) using the BWA software (https://bio-bwa.sourceforge.net/bwa.shtml, accessed on 2 July 2019) for subsequent variance analysis [44,45]. The SNPs and InDels were detected and filtered using GATK. The SNP/InDel index of the Euclidean distance (ED) and Δ(SNP/InDel-index) were calculated for all positions to determine the region associated with the mottled leaf.

### 4.7. QTL Analysis with Molecular Markers

Polymorphic InDel markers were developed in the candidate regions of mottled leaf based on the parent resequencing. InDel markers were designed with Primer Premier 5.0 (Table S2). PCR was carried out using 10 μL samples containing ~40 ng of genomic DNA, each primer at 0.5 μM, 200 μM dNTPs, 1× reaction buffer, and 0.5 U of Taq DNA polymerase (Aidlab Biotechnologies, Beijing, China). PCR amplification was performed using the following program: 94 °C for 5 min; 35 cycles of 94 °C for 30 s, 56 °C for 30 s, and 72 °C for 30 s; and 72 °C for 5 min [46,47]. The primers used in this study were synthesized using the BGI gene. Products were separated on an 8% polyacrylamide gel by electrophoresis. After electrophoresis at 220 V for 2 h, the gel was stained with 0.3% AgNO_3_ solution, and the silver-stained DNA bands were revealed.

### 4.8. Data Analysis

A, B, and H are homozygous ‘19’, homozygous ‘113’, and heterozygous F_1_ genotypes, respectively. We used JoinMap 4.0 to perform genetic mapping of F_2_ individuals using InDel markers on target chromosomes and obtained genetic distances. In R/qtl software (http://www.rqtl.org/), composite interval mapping (CIM) was used to test the significance of QTL [48,49]. The significance of each QTL was tested using LOD thresholds (*p* < 0.05), which were determined using 1000 permutations. For each detected QTL, a 2-LOD-support interval was calculated and defined by left and right markers. The QTLs were named according to chromosome locations and different environments.

### 4.9. Prediction of Candidate Genes and qRT-PCR

The gene ID, function, and structure data were obtained from the gourd genome database [44]. SoftBerry online software (http://linux1.softberry.com/, accessed on 22 November 2022) was used for gene structure prediction based on two family plants (*Arabidopsis*). The first, second, third, fifth, and seventh leaves under the growing tip at the 20-leaf stage of parents material and the first, third, and fifth leaves under the growing tip at the 20-leaf stage in the F_2_ population were selected to determine the expression of candidate genes. Total RNA was extracted using TRIzol reagent (Invitrogen, Carlsbad, CA, USA). Three pairs of primers for candidate genes were designed in total. Taq SYBR Green qPCR Premix (Yugong Biolabs, Inc., Jiangsu, China) was applied to perform qRT-PCR. The program used in this assay was as follows: 96 °C for 1 min; 30 cycles of 95 °C for 15 s, 56 °C for 15 s, and 72 °C for 45 s [50,51]. The Actin gene was used as the internal reference. Three technical replicates were set for each sample, and relative expression levels were quantified using the 2^−ΔΔCT^ method.

## 5. Conclusions

In summary, our findings indicate that the formation of mottled leaves did not affect the pigment content or photosynthesis in zucchini leaves. The large air space between cells in the palisade parenchyma was identified as the main factor underlying the formation of mottled leaf. During the early stages of leaf development, light is an important condition for the appearance of the mottled leaf. Genetic analysis revealed that mottled leaf is an inheritable quantitative trait and is controlled by multiple genes. Based on QTL-seq and the development of InDel markers, two major QTLs, *CpML1.1* and *CpML17.1*, were detected in different environments. They were located in a 925.2-kb interval on chromosome 1 and a 719.7-kb interval on chromosome 17, respectively. By performing gene annotation, gene sequence alignment, and qRT-PCR analysis, we found that the *Cp4.1LG01g23790* gene may be a candidate gene for mottled leaf in zucchini. Our results provide a suitable foundation for the fine mapping and mechanistic research of mottled leaf traits.

## Figures and Tables

**Table 1:** Microstructure analysis between ‘19’ and ‘113’.

Material	Leaf Thickness	Thickness of Upper Epidermis	Thickness of Palisade Parenchyma	Thickness of Sponge Parenchyma	Thickness of Lower Epidermis	CTR (%)	SR (%)
‘19’ mottle area	45.12 ± 0.09 a	4.64 ± 0.16 a	26.24 ± 0.10 a	8.69 ± 0.05 c	1.99 ± 0.10 a	58.15 ± 0.00 a	19.26 ± 0.00 c
‘19’ green area	45.09 ± 0.16 a	3.82 ± 0.08 b	26.22 ± 0.35 a	11.20 ± 0.06 a	2.32 ± 0.04 a	58.18 ± 0.01 a	24.83 ± 0.00 a
‘113’	43.66 ± 0.14 b	3.80 ± 0.04 bB	22.26 ± 0.20 b	10.24 ± 0.24 b	2.32 ± 0.41 a	50.98 ± 0.01 b	23.45 ± 0.01 b

Note: Values are the average ± SD on three replicates. Different letters indicate significant differences at the 0.05 level.

**Table 2:** QTL mapping of the mottled leaf trait in the F_2_ population.

QTL	Environment	Position Interval	Physical Distance (Kb)	LOD	Add	Dom	PVE (%)
CpML1.1	2019A	CHr01_18713051-CHr01_19638223	925.2	43.79	0.64	0.36	24.15
	2020A	CHr01_18590758-CHr01_19419173	828.4	31.18	0.56	0.04	23.82
	2020M	CHr01_18713051-CHr01_19638223	925.2	14.02	0.43	0.15	10.51
CpML17.1	2019A	CHr17_5225091-CHr17_5944838	719.7	38.70	0.63	0.03	20.80
	2020A	CHr17_5225091-CHr17_5944838	719.7	20.65	0.47	-0.01	16.25
	2020M	CHr17_5225091-CHr17_5944838	719.7	52.99	0.85	-0.19	38.68

## Data Availability

The raw datasets of QTL-seq generated during the current study are available in the NCBI repository, BioProject ID: PRJNA826826 (‘19’ and ‘113’) and PRJNA1077909 (M-pool and N-pool).

## Supplementary Materials

The supporting information can be downloaded at: https://www.mdpi.com/article/10.3390/ijms25052491/s1.
