# Supplementary material for: Morphological Analyses and QTL Mapping of Mottled Leaf in Zucchini (Cucurbita pepo L.)
Source: Int J Mol Sci. 2024 Feb 20;25(5):2491. doi: 10.3390/ijms25052491 (PMC10931640; doi:10.3390/ijms25052491)
Supplement: Supplementary file 1 [file ijms-25-02491-s001.zip › Figure S1.docx]

**Figure S1**. The protein sequences differences of *Cp4.1LG17g08260* between line ‘19’ and line ‘113’.
