# Supplementary material for: Morphological Analyses and QTL Mapping of Mottled Leaf in Zucchini (Cucurbita pepo L.)
Source: Int J Mol Sci. 2024 Feb 20;25(5):2491. doi: 10.3390/ijms25052491 (PMC10931640; doi:10.3390/ijms25052491)
Supplement: Supplementary file 1 [file ijms-25-02491-s001.zip › Table S1.docx]

**Table S1. Descriptive statistics for mottled leaf trait in F_2_ population**

| Enviroment | 19 | 113 | | F_1_ | F_2_ population | | | | |
| --- | --- | --- | --- | --- | --- | --- | --- | --- | --- |
|  |  |  |  |  | Skewness | Kurtosis | Average | SD | CV(%) |
| 2019A | 3 | 0 | 2 | | −0.69 | −0.863 | 1.596 | 0.993 | 63.29 |
| 2020A | 3 | 0 | 2 | | 0.286 | −0.981 | 1.281 | 1.001 | 78.14 |
| 2020M | 3 | 0 | 2 | | 0.330 | −0.901 | 1.313 | 0.989 | 75.32 |
