# Supplementary material for: Morphological Analyses and QTL Mapping of Mottled Leaf in Zucchini (Cucurbita pepo L.)
Source: Int J Mol Sci. 2024 Feb 20;25(5):2491. doi: 10.3390/ijms25052491 (PMC10931640; doi:10.3390/ijms25052491)
Supplement: Supplementary file 1 [file ijms-25-02491-s001.zip › Table S2.docx]

**Table S2. InDel primer information**

| Molecular marker name | Forward primer sequence | Reverse primer sequence |
| --- | --- | --- |
| CHr01_16983010 | TTAGGAGGAAATGGAAGGAG | GAACGAGTGTCAGCGAGG |
| CHr01_17302997 | GCTGTCCTATCACTGCCTA | TAGAGGTCGGGAGTTCAA |
| CHr01_17745190 | AGGGAGTGGACACAAGATGAT | TACCACCTGTTCACTTCTCTAATC |
| CHr01_18154650 | GATGGTGGAAAGGCGAC | AAGGAATGTTGTTTGGTCAGT |
| CHr01_18590758 | GTGTGTTCTTCGTTTGGCAT | GCACACACTTCTATGAGGGC |
| CHr01_18713051 | ATAATACCGCCACGAACCA | CAACAAGTGGAAGGCAAGTAG |
| CHr01_18955752 | CTGTTCTTCCCACCGTTTG | CAAGCATACAACCACCACGA |
| CHr01_19094186 | CTCTTTCGTCCCTCATCGG | TCTCCGCCCTTCGTATCTC |
| CHr01_19419173 | TCCTCTCCTCCTACCCGTGT | TGTGACATCCTGGTTCAGTTTAG |
| CHr01_19638223 | TTGTTTGTATTGAGTATGAATGCTT | AGGCGTTTGTCAAGGTTATG |
| CHr01_19745132 | TAAGAGATTCAATGTTTGTCTACGA | TTTTCCCGTTTGAGGTGG |
| CHr01_20164367 | GTCAGCCAGCAGCAGAG | AGAGATTAGAGGTGAAGCAAA |
| CHr01_20395706 | GCTGATTCCCAAAGGGTAGA | TGGTGTGCCATTATCATTGTG |
| CHr01_21277261 | CCACTTTCATCCACTAACGC | GTGTATGCCAGGTTGAGAGC |
| CHr17_4323795 | CTGAGCAGGAATGGTGAGC | TGTTTGATGATTGATGGGGT |
| CHr17_4634475 | GGTATCTTTTTGGCGGGTT | GAACAATACAACACAGAAACGACAG |
| CHr17_4951599 | AAACTGATAGGCTGATGACGAT | ACCCATTATGTATCGTTCCACT |
| CHr17_5038382 | TTCTCTCCGCTTCATCATCG | CGAGCAGTTCAGTCCAATAAATG |
| CHr17_5225091 | AAGAGGACTTTGCTTTGGACT | TTGAGGATTATTGGGGTGTG |
| CHr17_5303229 | AAATCCAAACTGCCCGTG | CCCACAGTTGATGAGCAGAG |
| CHr17_5465890 | GATACAATCATCCAACTTCCACG | AAGGCTGCTTTATGGGGTC |
| CHr17_5581523 | TTGAACCCGAACCAGACC | CAGCCTCTACTACAACACTACCTTT |
| CHr17_5944838 | TTTATGTGGGACCTTCAACTTC | GAATCTCTCCTTGTCATAGGCATCC |
| CHr17_6230275 | ACCTCCACACGGTATTGCTC | CCAAGAAACTAGCTTTGTGACC |
| CHr17_6546502 | GGGAGTGGATTGTGAGGTCT | CAAATCTCTTCTTTATGACACCAG |
