# Supplementary material for: Morphological Analyses and QTL Mapping of Mottled Leaf in Zucchini (Cucurbita pepo L.)
Source: Int J Mol Sci. 2024 Feb 20;25(5):2491. doi: 10.3390/ijms25052491 (PMC10931640; doi:10.3390/ijms25052491)
Supplement: Supplementary file 1 [file ijms-25-02491-s001.zip › Table S3.docx]

**Table S3. Candidate genes information in *CpML1.1* region**

| Gene ID | CDS | | Promoter | UTR | Position | Function | |
| --- | --- | --- | --- | --- | --- | --- | --- |
| *Cp4.1LG01g24790* | | 1SNP | 2InDel | 3SNP | 18,739,766 - 18,743,406 | | SEC12-like protein 1 |
| *Cp4.1LG01g24570* | | 2SNP | 4InDel/1SNP | 3InDel | 18,946,606 - 18,950,012 | | S-acyltransferase |
| *Cp4.1LG01g24580* | | 5SNP | 13InDel/21SNP | 8SNP/2InDel | 18,950,889 - 18,958,374 | | Transmembrane amino acid transporter family protein |
| *Cp4.1LG01g24550* | | 13SNP | 6InDel/11SNP | 5InDel/49SNP | 18,963,791 -18,977,640 | | ABC transporter G family-like protein |
| *Cp4.1LG01g24420* | | 1SNP | 12InDel/29SNP | 4InDel/4SNP | 19,094,037 - 19,101,228 | | Nuclear receptor corepressor 1 |
| *Cp4.1LG01g24300* | | 7SNP | 2InDel/5SNP | 4SNP | 19,152,530 -19,155,006 | | Pentatricopeptide repeat-containing protein, putative |
| *Cp4.1LG01g24140* | | 1SNP | 4InDel/17SNP | 0 | 19,235,552 -19,236,619 | | Plant protein 1589 of unknown function |
| *Cp4.1LG01g24080* | | 3SNP | 10InDel/24SNP | 2InDel/7SNP | 19,238,463 -19,245,009 | | Phosphatidylinositol N-acetyglucosaminlytransferase subunit P-related |
| *Cp4.1LG01g24120* | | 1InDel/13SNP | 3SNP | 1InDel/5SNP | 19,257,200 -19,261,989 | | Unknown protein |
| *Cp4.1LG01g24100* | | 7SNP | 6InDel/7SNP | 2InDel/9SNP | 19,263,180 -19,269,689 | | Pax6 |
| *Cp4.1LG01g24070* | | 1SNP | 12InDel/16SNP | 3SNP | 19,270,377 -19,276,869 | | SET domain-containing protein |
| *Cp4.1LG01g23790* | | 1InDel/7SNP | 14InDel/22SNP | 5InDel/10SNP | 19,473,360 - 19,476,725 | | TPX2 (Targeting protein for Xklp2) family protein |
