# Supplementary material for: Morphological Analyses and QTL Mapping of Mottled Leaf in Zucchini (Cucurbita pepo L.)
Source: Int J Mol Sci. 2024 Feb 20;25(5):2491. doi: 10.3390/ijms25052491 (PMC10931640; doi:10.3390/ijms25052491)
Supplement: Supplementary file 1 [file ijms-25-02491-s001.zip › Table S4.docx]

**Table S4. Candidate genes information in *CpML17.1* region**

| Gene ID | CDS | | Promoter | UTR | Position | Function |
| --- | --- | --- | --- | --- | --- | --- |
| *Cp4.1LG17g08460* | | 1InDel/4SNP | 8InDel/12SNP | 3InDel/ | 5,286,872- 5,297,242 | Myosin family protein, putative, expressed |
| *Cp4.1LG17g08410* | | 1 SNP | 0 | 0 | 5,329,112 - 5,329,477 | Oxidoreductase/transition metal ion-binding protein |
| *Cp4.1LG17g08430* | | 2InDel/4SNP | 3InDel | 2SNP | 5,359,725 - 5,363,671 | DNA-binding bromodomain-containing family protein |
| *Cp4.1LG17g08260* | | 2SNP | 9InDel/20SNP | 2InDel/17SNP | 5,391,015 -5,395,142 | TRICHOME BIREFRINGENCE-LIKE 14 |
| *Cp4.1LG17g08270* | | 2SNP | 2InDel/1SNP | 5InDel/7SNP | 5,401,552 - 5,403,430 | 60S ribosomal protein L6 |
| *Cp4.1LG17g08290* | | 1SNP | 10InDel/ | 2InDel/ | 5,404,299 - 5,408,656 | Chaperone protein DNAj, putative |
| *Cp4.1LG17g08310* | | 6SNP | 20InDel/20SNP | 5SNP | 5,410,906 - 5,414,530 | Pathogenesis-related thaumatin family protein |
| *Cp4.1LG17g08280* | | 1SNP | 2SNP | 0 | 5,430,328 - 5,433,193 | tRNA (Cytidine(34)-2'-O)-methyltransferase |
| *Cp4.1LG17g08140* | | 3SNP | 26SNP | 2SNP | 5,482,989 - 5,487,173 | E3 ubiquitin ligase BIG brother-like protein |
| *Cp4.1LG17g08060* | | 3SNP | 4InDel/8SNP | 1InDel | 5,494,602 - 5,499,665 | Protein DETOXIFICATION |
| *Cp4.1LG17g08130* | | 1SNP | 1SNP | 0 | 5,508,757 - 5,515,844 | Protein FRA10AC1 |
| *Cp4.1LG17g08300* | | 1SNP | 2InDel | 3InDel/3SNP | 5,377,093 - 5,390,586 | Beta-glucosidase |
| *Cp4.1LG17g08250* | | 3SNP | 7InDel/13SNP | 1InDel/10SNP | 5,370,182 - 5,373,885 | Box C/D snoRNA protein 1 |
| *Cp4.1LG17g08400* | | 2SNP | 0 | 0 | 5,316,218 - 5,316,673 | Unknown function |
| *Cp4.1LG17g08470* | | 4SNP | 2InDel | 2InDel/3SNP | 5,340,761 - 5,343,901 | Unknown function |
